# Electrolytic Bubble Coalescence on Hydrophobic Cavity Arrays Determines Departure Radius and Lowers Electrolyte Supersaturation

**DOI:** 10.1002/smll.202505728

**Published:** 2025-09-18

**Authors:** Akash Raman, Stefan Schlautmann, Han Gardeniers, David Fernández Rivas

**Affiliations:** ^1^ Mesoscale Chemical Systems Group, MESA+ Institute for Nanotechnology, Faculty of Science and Technology University of Twente P.O. Box 217, 7500 AE Enschede The Netherlands

**Keywords:** bubble coalescence, bubble dynamics, electrolytic bubbles, hydrogen bubbles, water electrolysis

## Abstract

Bubble evolution on electrodes is a complex process that begins with the stochastic nucleation of bubbles on the electrode surface, followed by bubble growth due to diffusion and coalescence, and bubble departure. The stochasticity of bubble evolution on conventional electrodes is a significant challenge in efforts to study electrolytic bubbles. In this investigation, the growth of electrolytic hydrogen bubbles is studied on microfabricated silicon electrodes with arrays of hydrophobic cavities. These hydrophobic pits act as preferential nucleation sites for bubbles—thus lowering the degree of spatial‐randomness in bubble nucleation and enabling the study of bubbles growing in the presence of coalescence with greater control. Substrates with different spacings between the hydrophobic pits were fabricated. It is shown that coalescence with neighboring bubbles strongly determines the departure radius of bubbles. Further analysis of the bubble growth rate and electrode coverage indicates that closer pits decrease the electrolyte supersaturation while increasing electrode coverage.

## Graphical abstract

Bubble evolution is a key source of inefficiency in electrolyzers. In this study, custom‐fabricated electrodes with arrays of preferential nucleation sites are used to overcome the spatial randomness of bubble nucleation. Bubble departure size is shown to strongly depend on coalescence. Furthermore, increasing nucleation site spacing is shown to increase the concentration of dissolved gas in the electrolyte.

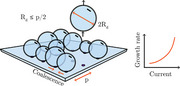

## 1 Introduction

Bubbles are generated in a number of key electrochemical reactions which result in the formation of gaseous products such as the chlor‐alkali process and water electrolysis.^[^ ^1^, ^2^, ^3^ ^]^ These electrolytic bubbles can decrease the efficiency of electrochemical reactions by blocking the electrode surface and rendering portions of the electrode inactive,^[^ ^4^, ^5^ ^]^ and can lead to hyperpolarization of the electrode.^[^ ^6^, ^7^ ^]^ Electrolytic bubbles cause additional Ohmic losses by impeding ion conduction in the electrolyte.^[^ ^3^ ^]^ On the other hand, bubbles are also known to induce convection in the vicinity of the electrode during growth and upon departure—which improves mass transfer^[^ ^8^ ^]^ and also exhibit a concentration lowering effect which alleviates the concentration overpotential.^[^ ^9^, ^10^, ^11^ ^]^ A key first step in improving the efficiency of electrochemical reactions is to gain a better understanding of the precise behavior and effects of bubbles in electrochemical systems.

In gas‐evolving electrochemical reactions, the reaction products are initially dissolved in the electrolyte and often have low solubilities.^[^ ^12^ ^]^ Bubble nucleation occurs when a threshold saturation concentration is reached. These bubble nuclei grow by absorbing dissolved gas from the surrounding saturated electrolyte. Nucleation in electrochemical processes is typically heterogeneous, i.e., it occurs at the electrode‐electrolyte interface.^[^ ^3^, ^13^, ^14^ ^]^ The creation of the gas‐liquid phase boundary in homogeneous nucleation requires a large amount of energy and is only known to occur in cases such as boiling liquids.^[^ ^15^ ^]^ On the other hand, heterogeneous nucleation is more common because the thermodynamic barrier for the formation of the gas‐phase is lowered by the presence of a solid surface.^[^ ^13^, ^14^, ^16^ ^]^

The literature on electrolytic bubble evolution can be broadly classified into those concerning micro‐electrodes and those concerning macro‐electrodes. On commercially used electrodes, such as meshes and foams,^[^ ^17^, ^18^, ^19^, ^20^, ^21^ ^]^ bubble nucleation is uncontrolled and results in the simultaneous nucleation and growth of bubbles throughout the electrode.^[^ ^22^ ^]^ The direct observation of bubble‐related physical phenomena such as micro‐convective flows^[^ ^8^ ^]^ with high spatio‐temporal resolution on such macro‐electrodes is challenging.^[^ ^23^ ^]^

In contrast, some studies have focused on bubble evolution on micro‐ and nano‐electrodes.^[^ ^24^, ^25^, ^26^, ^27^, ^28^, ^29^ ^]^ These microelectrodes studies have offered valuable insight into bubble growth dynamics^[^ ^30^, ^31^, ^32^ ^]^ the role of the electrolyte,^[^ ^33^, ^34^ ^]^ the role played by Marangoni effects^[^ ^35^, ^36^, ^37^, ^38^, ^39^ ^]^ and buoyancy‐driven flows.^[^ ^40^ ^]^ Nonetheless, the translation of results from microelectrodes to commercially‐relevant porous electrodes, or even to larger planar electrodes remains a challenge.^[^ ^41^ ^]^ One specific challenge in the scaling up of results from microelectrode studies is the absence of next‐neighbor bubble coalescence.

In summary, gas‐evolving macro‐electrodes present complex experimental challenges in capturing the intricacies of fluid and mass transfer and results from microelectrodes studies need to be re‐validated in larger systems. Therefore, a meso‐scale approach is required wherein bubble evolution is studied on small enough systems where experimental techniques of sufficient spatio‐temporal resolution can be utilized while retaining spatial control over bubble nucleation sites.

Previously, hydrophobic spots^[^ ^42^ ^]^ and hydrophobic cavities^[^ ^10^, ^43^, ^44^, ^45^ ^]^ have been included on gas‐evolving electrodes as a method of controlling the site of bubble nucleation.^[^ ^46^ ^]^ The hydrophobic cavities used by Raman et al.^[^ ^9^, ^10^ ^]^ contain stable gas bubbles even before the start of electrolysis. The presence of such gas cavities is known to significantly lower the threshold saturation required for subsequent bubble nucleation during electrolysis.^[^ ^13^ ^]^ As a result, these hydrophobic cavities act as preferential nucleation sites for gas bubbles. In addition, the force of surface tension which pins bubbles to such hydrophobic cavities is determined by the length of the contact line i.e., the circumference of the cavity. Therefore, hydrophobic cavities additionally offer a way to control the bubble departure radius.^[^ ^9^, ^43^ ^]^

In this study, bubble evolution is studied on microfabricated silicon‐based electrodes with ordered arrays of hydrophobic cavities. The growth of multiple bubbles in close proximity on this electrode closely mimics bubble growth on conventional macro‐electrodes but with greater spatial control over bubble nucleation. This approach represents a significant advance over the previous use of hydrophobic cavities in model (micro‐)electrode systems. Furthermore, this electrode architecture acts as a model system to test the applicability of hydrophobic cavities and other nucleation site control methods as tools for bubble management in electrolyzers.

## 2 Results and Discussion

### 2.1 Experimental Setup

**Figure 1:**
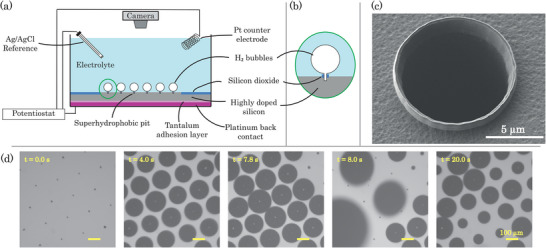
a) Schematic diagram of the lateral‐view of the three‐electrode electrolysis cell used in this study. A constant current is applied between the microfabricated silicon substrates and a platinum coil counter‐electrode. Bubbles preferentially nucleate at hydrophobic pits etched into the silicon substrate. Panel (b) shows a close‐up of a single hydrophobic pit with a bubble pinned to it. The bubbles pin to raised silicon oxide rings which encircle the hydrophobic pits. Panel (c) shows a close‐up SEM image of a single hydrophobic pit. The silicon oxide ring is clearly seen protruding from the silicon surface. d) Sequence of optical images from an experiment with a pit separation ratio *p* = 0.241 with an electrolysis current of *i* = 1000 μA. The array of hydrophobic pits is visible in the first image as black dots against the brighter silicon substrate. The panel corresponding to *t* = 7.8 s and *t* = 8.0 s show bubbles just before and just after a coalescence event. The final sub‐panel corresponding to *t* = 20.0 s shows asynchronous bubble growth.

**Figure** **1** shows a schematic representation of the experimental setup used in this study. The experiments were conducted on microfabricated silicon electrodes with triangular arrays of hydrophobic cavities. A detailed description of the electrode fabrication steps is provided in the Experimental Section. The microfabricated electrode were washed with iso‐propyl alcohol, water (Milli‐Q 18.2 MΩcm) and dried in air. Then, the electrodes were placed in a custom built 3‐D printed electrode holder with an angled port to position a low profile Ag/AgCl (Pine Research RRPEAGCL) reference electrode in a repeatable manner.^[^ ^9^ ^]^ A platinum coil (Basi MW‐1033) was used as the counter electrode and was immersed into the electrolyte at open‐top of the holder where the electrolyte was in contact with the air at all times. Connections to the working electrode sample were achieved through the use of a plunger screw at the bottom of the holder which made contact with the back contact terminal of the samples. Since the exposed silicon electrode would etch in alkaline solutions, 0.1 M H_2_SO_4_ (96% Sigma Aldrich, prepared in 18.2 MΩcm Milli‐Q water) was used as electrolyte in all experiments. In order to promote the trapping of gas in the hydrophobic cavities on the electrodes, the electrolyte was not degassed before experiments and remained in equilibrium with the atmosphere in the laboratory.

The exposed electrode area (boron‐doped silicon) was a 3 × 3∼mm^2^ area at the center of each 10 × 10 mm^2^ sample. Note that the geometric area of the silicon electrode that is active for the hydrogen evolution reaction is less than 9 mm^2^ since the pits occupy a finite area and are not sites of Faradaic gas generation. Table S1 (Supporting Information) lists the actual current densities for each pit spacing and applied current.

The three‐electrode system was connected to a potentiostat (BioLogic SP‐150) and constant currents *i* = 100, ∼500, ∼1000, ∼2500, and 5000 μA were applied to the terminals. Four pit spacings were considered which are referenced by their pit‐spacing ratio *p*/2*R*_*f*_ = 0.108, ∼0.241, ∼0.539, and 1.206 where *p* is the distance between two adjacent pits on the substrates and *R*_*f*_ is the theoretical departure radius or the Fritz radius. *R*_*f*_ is defined by balancing the force of surface tension and buoyancy acting on the bubbles:^[^ ^47^ ^]^

(1) $$R_{f}={\left(\frac{3\sigma r_{p}}{2\Delta \rho g}\right)}^{\frac{1}{3}}$$

where *r*_*p*_ is the pit radius, σ = 72.414 × 10^−3^ N/m is the interfacial surface tension of hydrogen in 0.1 M H_2_SO_4_,^[^ ^48^ ^]^ Δρ = 997 kg/m^3^ is the density difference between hydrogen gas and water and *g* = 9.81 m/s^2^ is gravitational acceleration (see Table S2, Supporting Information, for values of physical parameters).

The sample holder containing the sample and the electrodes was placed under a optical microscope (Olympus BXFM) with a digital camera (PointGrey Flea3) attached to it to record experiments in a top‐down view as shown in Figure 1. The camera recorded image sequences at 10 fps in all experiments. A 5× objective was used for *p*/2*R*_*f*_ = 0.108 and 0.241 and a 2× objective was used for *p*/2*R*_*f*_ = 0.539 and 1.206. A lower magnification was used for the substrates with larger pit spacings in order to capture as many pits as possible within the field‐of‐view of the optical system. The optical resolutions of the experimental images (1552 × 1552 pixels, 8‐bit grayscale) recorded at 2× and 5 × magnification are ∼ 0.5 μm/px and ∼1.75 μm/px respectively. It is worth noting that for the lower magnification nearly the entire electrode area was imaged whereas for the higher magnification (for smaller pit spacings), ≈ 25 % of the electrode was imaged. The number of pits visible in the field of view for each spacing is presented in Section S9 (Supporting Information).

Bubble radii *R*_*b*_ were extracted from the recorded camera frames through a two‐stage circule detection algorithm^[^ ^49^, ^50^ ^]^ and the growth of bubbles was tracked across frames. The nucleation time of each bubble, *t*_0_, was estimated by linear interpolation of the first 1.5 s of $R_{b}^{2}$ and *t* data points, where *t* is experimental time. A detailed description of image processing steps and data analysis are provided in Section S2 (Supporting Information).

### 2.2 Bubble Growth Dynamics

**Figure 2:**
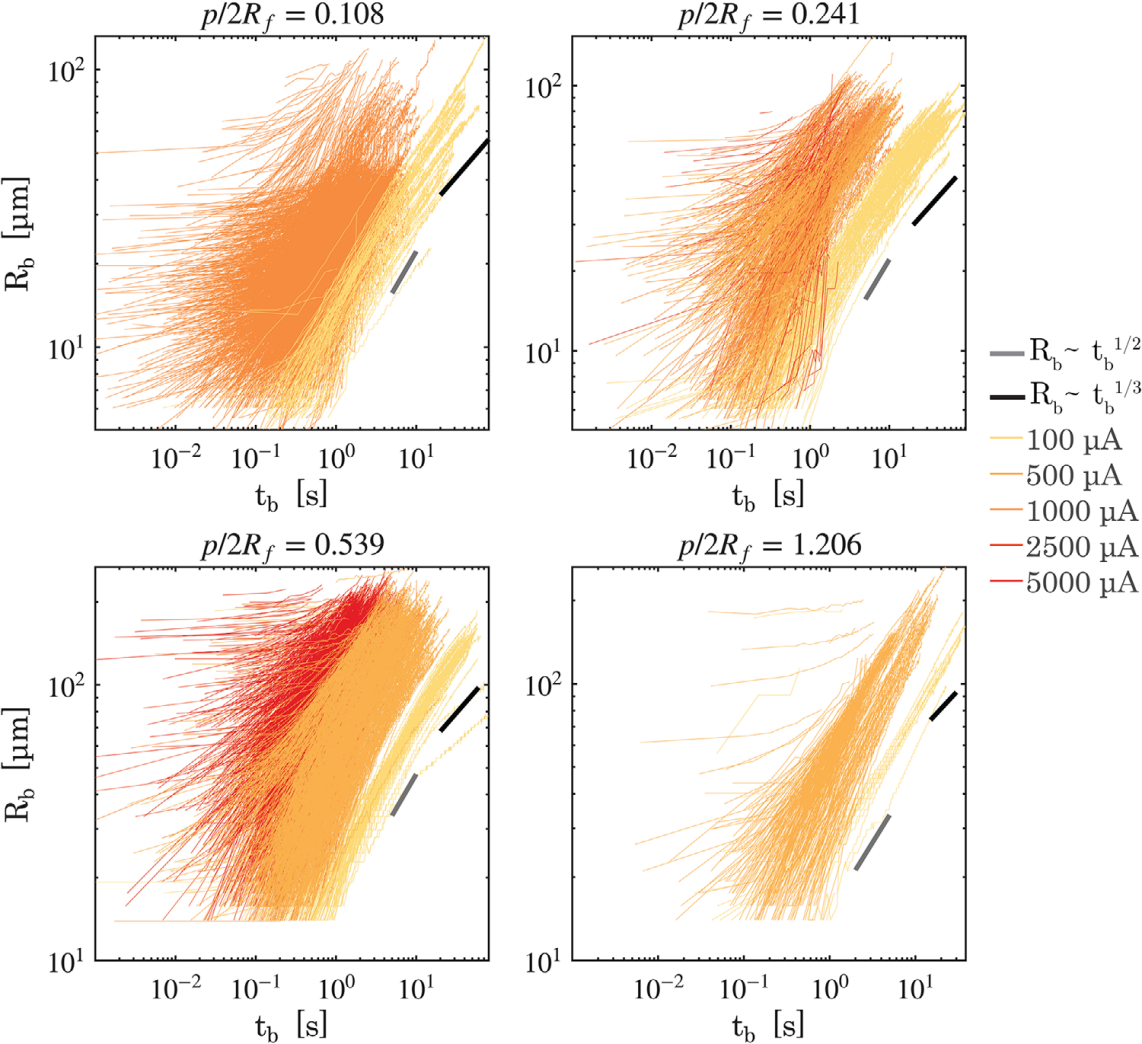
The bubble growth curves of bubbles, i.e., bubble radii *R*_*b*_ plotted as functions of bubble lifetime *t*_*b*_, are shown on a log‐log scale. Growth curves beginning at *R*_*b*_ ⩾ 20 μm represent bubbles resulting from the coalescence of preceding bubbles. The gray line indicates the slope corresponding to $R_{b}\propto t_{b}^{1/2}$ and the black line indicates the slope corresponding to $R_{b}\propto t_{b}^{1/3}$.

**Figure** **2** shows the bubble radius *R*_*b*_ plotted against the bubble lifetime *t*_*b*_ for bubbles driven by different electrolysis currents *i* shown on a logarithmic scale (see Figure S3, Supporting Information). The data is grouped into four subplots showing data from substrates with the four different pit‐spacing ratios *p*/2*R*_*f*_ used in the study. Bubble growth is commonly described by a power‐law relationship between *R*_*b*_ and *t*_*b*_,

(2) $$R_{b}=\beta t_{b}^{\alpha }$$

where α and β are the time‐exponent and the bubble growth coefficient respectively. The value of α is known to depend on the mechanism of mass‐transfer into the bubble. The three commonly reported bubble growth modes—the inertial (or pressure‐driven), diffusion‐limited, and reaction‐limited (or supply‐limited) growth regimes correspond to α = 1, 1/2 and 1/3 respectively.^[^ ^9^, ^10^, ^29^, ^32^, ^44^, ^51^, ^52^, ^53^ ^]^

In logarithmic representations of bubble growth curves, the values of α and log β are the slope and intercept respectively, and the bubble growth equation becomes:

(3) $$\log R_{b}=\log\beta +\alpha \log t_{b}$$

Straight lines with slopes corresponding to α = 1/2 (diffusion‐limited growth) and α = 1/3 (reaction‐limited growth) are shown in Figure 2. In the figure, it can be seen that the initial slopes of the growth curves for all pit spacing ratios correspond to the diffusion‐limited regime (α = 1/2). This justifies the choice of using the equation $R_{b}\propto t_{b}^{1/2}$ to estimate the nucleation time of bubbles.

As the bubbles grow larger, bubble growth transitions to a reaction‐limited growth regime and the slopes of the bubble growth curves match α = 1/3 on substrates with *p*/2*R*_*f*_ = 0.108, 0.241, and 0.539. Notably, this transition is absent in the growth curves of bubbles growing on substrates with the pit‐spacing ratio *p*/2*R*_*f*_ = 1.206 where the slopes of the bubble growth curves remain closer to α = 1/2. The transition from diffusion‐limited growth to reaction‐limited growth is determined by the size of the bubble relative to the size of the electrode it grows on. Bubbles growing on substrates with *p*/2*R*_*f*_ = 0.539 and 1.206 grow to the larger sizes. Furthermore, these substrates contain fewer hydrophobic cavities which translates to a greater effective electrode area per cavity.

Apart from the bubble growth regimes, the following observations can be made from Figure 2. First, bubbles driven by greater currents grow faster and depart from the electrode sooner.

Secondly, the error in the estimation of *t*_0_ is vast in cases of bubbles that start from large *R*_*b*_ such as those which are formed as a result of bubble coalescence. The assumption of initial diffusion‐limited growth does not hold for those bubbles.

Finally, it is evident that there is a wide spread in the bubble departure radii as well as the bubble growth rates even when comparing at the same electrolysis current. Thus, both these parameters warrant further analysis.

### 2.3 Bubble Departure Radii

**Figure 3:**
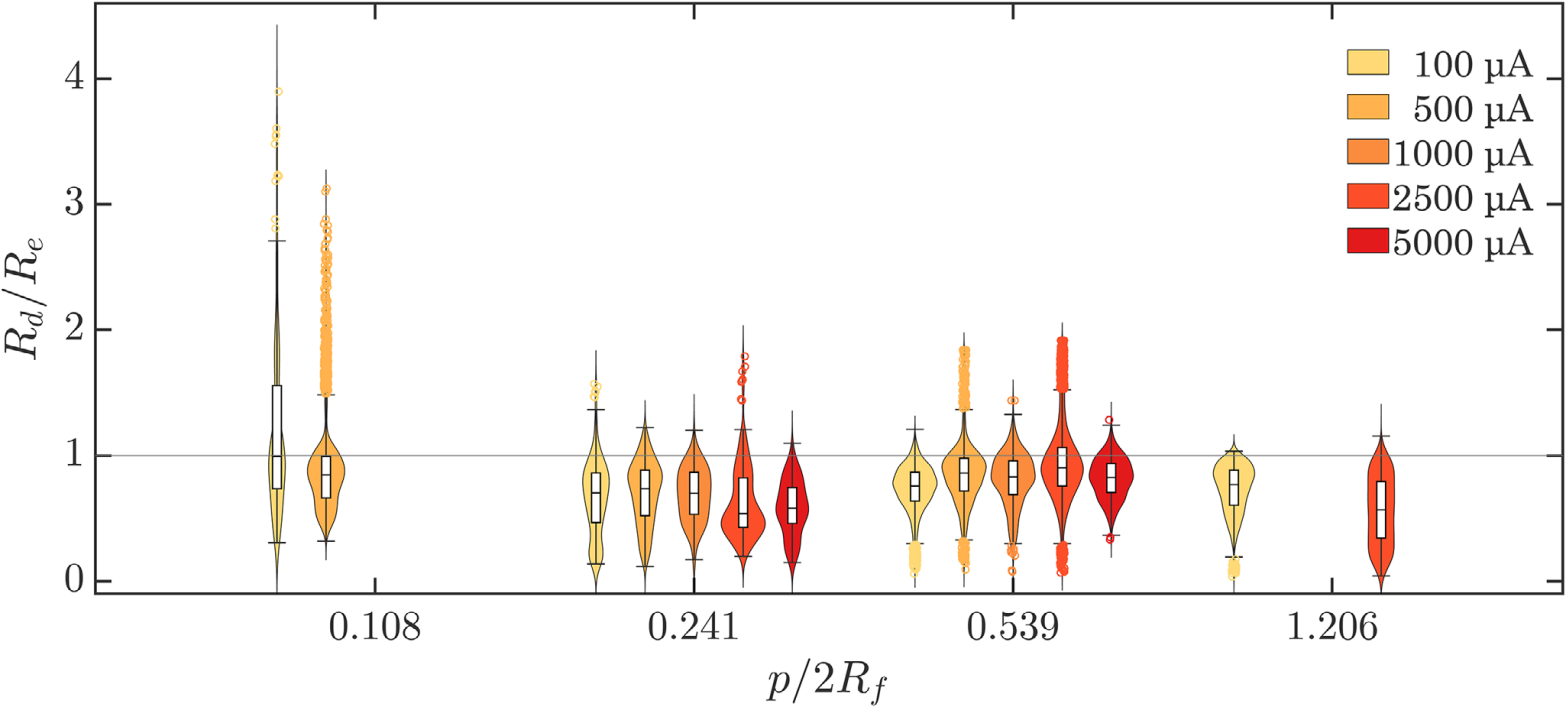
Violin and box plots of the normalized departure radii, *R*_*d*_/*R*_*e*_ of bubbles growing on substrates where *R*_*d*_ is the radius of bubbles at the time of their departure and *R*_*e*_ is the expected departure radius (see Equation 4). Data for bubbles driven by different currents are shown by the color of the kernel density estimation curves and grouped by the pit‐separation ratios *p*/2*R*_*f*_ of the substrates, where *p* is the distance between adjacent pits and *R*_*f*_ is the Fritz departure radius. The horizontal black line represents *R*_*d*_/*R*_*e*_ = 1. The bandwidths were estimated using the normal‐approximation method. The white boxes represent the middle 50 % of each dataset and show the sample median as a horizontal line. The whiskers represent the extent of data and outliers are plotted as circles.

**Figure** **3** shows violin and box plots of the bubble departure radii of bubbles (*R*_*d*_) normalized by their expected departure radius, *R*_*e*_ (see Equation 4 below). The departure radius, *R*_*d*_ is taken to be the radius of a bubble at the end of its observable lifetime on the electrode. The data in the plot is grouped by the pit‐spacing ratio of the substrates and the electrolysis current applied. The pit‐spacing ratio *p*/2*R*_*f*_ is determines the dominant mechanism of bubble departure associated with the substrate. For *p*/2*R*_*f*_ ⩾ 1, bubbles growing at adjacent pits reach the Fritz radius before coalescing with any neighboring bubbles. Whereas, for *p*/2*R*_*f*_ < 1 adjacent bubbles will coalesce before they become large enough to depart due to buoyancy alone. Therefore, the expected departure radius, *R*_*e*_ can be described as

(4) $$R_{e}=\left\{\begin{array}{c} p/2\;\text{if}\;p/2R_{f}<1\\ R_{f}\;\text{if}\;p/2R_{f}\geq 1 \end{array}\right.$$

Therefore, in an ideal system, where all bubbles depart at the expected bubble departure radius, *R*_*e*_, the normalized bubble departure radius of all bubbles will be *R*_*d*_/*R*_*e*_ = 1 (indicated by the horizontal black line in Figure 2.3). The same plots are presented in Figure S6 (Supporting Information) without normalization. Several key observations can be made from the violin and box plots presented in Figure 2.3.

First, the sample medians of *R*_*d*_/*R*_*e*_ for all pit spacing ratios and currents considered in the study are <1. This indicates that the vast majority of bubbles (83.39% of bubbles, see S5, Supporting Information) depart at *R*_*d*_ ⩽ *R*_*e*_. This confirms that bubble coalescence is the dominant mechanism responsible for bubble departure on these substrates.

Second, for all pit spacing ratios and currents, a minority of bubbles are seen departing at radii >*R*_*e*_. Such bubbles likely continued growing after coalescence with neighboring bubbles and did not detach from the surface. Bubble detachment after coalescence occurs in two cases—(i) if the radius of the resulting bubble is >*R*_*f*_ or, (ii) if the coalescence causes a strong surface wave which overcomes the pinning force at the three‐phase boundary.^[^ ^54^, ^55^ ^]^ In the first case, the force of buoyancy experienced by the resulting bubble exceeds the force of surface tension pinning the bubble to the surface. In the latter case, the surface wave which propagates across the bubble surface as the excess surface energy is dissipated, is strong enough to cause detachment at radii <*R*_*f*_.^[^ ^54^ ^]^ Both these modes of detachment become more likely as the sizes of the parent bubbles increases. The radius at which bubbles coalesce increases with increasing pit spacing ratio. This explains why the largest bubbles grow up to 4 × *R*_*e*_ on substrates with *p*/2*R*_*f*_ = 0.108.

Finally, a wide distribution of *R*_*d*_/*R*_*e*_ is seen for all pit‐spacing ratios and current considered in the study. This spread of data can be explained through three main factors.

First, the nucleation of bubbles is not (temporally) synchronized at the onset of electrolysis (as shown in Figure 1d and Video S1, Supporting Information), i.e., bubbles nucleate at different locations on the electrodes at different times. This inherent experimental asynchronicity in bubble nucleation leads to asymmetric bubble coalescence, i.e., coalescence between bubbles of dissimilar sizes.

Second, despite the presence of the hydrophobic cavities, bubbles occasionally nucleate at locations other than the pits (see Figure S4 and Video S1, Supporting Information). This may be due the presence of debris on the electrodes, or due to localized roughness resulting from minor variations during the fabrication process.

Thirdly, some hydrophobic cavities were observed to be “inactive” (see Figure S4 and Video S1, Supporting Information) i.e., they no longer acted as preferential nucleation sites for the bubbles. The deactivation of similar nucleation sites in the case of acoustic bubbles is known to occur due the liquid jetting during bubble collapse.^[^ ^56^ ^]^ It is possible that similar liquid jets caused by the breakup gas‐bubble interface during bubble departure results in the complete wetting of the hydrophobic pits. This requires further study which is beyond the scope of this work.

### 2.4 Bubble Growth Rate

The volumetric bubble growth rate, $\dot{V}$ can be calculated as:

(5) $$\dot{V}=\frac{4}{3}\pi \frac{dR_{b}^{3}}{dt_{b}}$$

We normalize the volumetric bubble growth rate by the volumetric rate of gas generation at the electrode surface to obtain the non‐dimensional bubble growth rate, ${\dot{V}}^{\prime }$,

(6) $${\dot{V}}^{\prime }=\frac{\dot{V}}{\frac{iRT}{zFP}}$$

where R is the universal gas constant, F is the Faraday's constant, z is the number of electrons transferred per molecule of hydrogen produced and P is the ambient pressure. Naturally, bubbles driven by greater electrolysis currents grow faster. Therefore, normalization with the rate of gas generation (which depends on *i*) allows for the direct comparison of the growth rates of bubbles driven by different currents.

**Figure 4:**
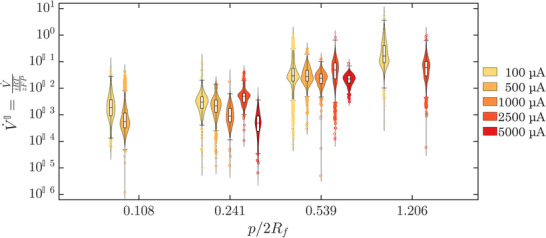
Violin and box plots of ${\dot{V}}^{\prime }$, the bubble growth rate normalized by the molar rate of hydrogen production at the electrode surface. Data for bubbles driven by different currents are shown by the color of the kernel density estimation curves and grouped by the pit‐separation ratios *p*/2*R*_*f*_ of the substrates, where *p* is the distance between adjacent pits and *R*_*f*_ is the Fritz departure radius. The bandwidths were estimated using the normal‐approximation method. The white boxes represent the middle 50% of each dataset and contain the sample median shown as a horizontal line. The whiskers represent the extent of data falling within 1/5 × the interquartile range and outliers are plotted as circles. It can be seen that ${\dot{V}}^{\prime }$ increases with increasing pit‐separation ratios.

**Figure** **4** shows box and violin plots of the normalized bubble growth rate, ${\dot{V}}^{\prime }$ of bubbles driven by different *i*, growing on electrodes with different pit‐spacing ratios. See Section S9 (Supporting Information) for a similar box and violin plot of bubble growth rate $\dot{V}$ without normalization.

Bubble growth is driven by the diffusive transport of dissolved gas from the electrolyte and the bubble growth rate increases with increasing gas supersaturation in the electrolyte.^[^ ^44^ ^]^ Therefore, trends in Figure 2.4 can be related to the effective supersaturation of hydrogen experienced by bubbles in different experiments. A clear dependence of the bubble growth rate on the pit spacing ratio *p*/2*R*_*f*_ can be seen in Figure 2.4. The normalized bubble growth rate of bubbles decreases with decreasing *p*/2*R*_*f*_ for all currents. This indicates that for the same applied current, bubbles growing on substrates with smaller pit spacing ratios experience lower supersaturation. Bubbles growing on substrates with smaller pit depart at smaller bubble radii as discussed in the previous section. Therefore, for the same applied current, bubbles depart more frequently on substrates with smaller pit spacing ratios. This bubble‐departure driven disruption of the concentration boundary layer leads to a lower gas supersaturation in the electrolyte in the vicinity of the electrode. This has significant implications for the efficiency of electrolysis and demonstrates a mechanism through which bubbles can alleviate the concentration overpotential during electrolysis.

It can be seen in Figure 4 that upon normalization, no current‐dependent trend appears in the bubble growth rate data. This demonstrates that, for the current densities applied in this study, the effect of current was a direct, linear increase in the growth rate which was removed upon normalization. This further indicates that physical phenomena which strengthen with increasing electrolysis such as thermal Marangoni effects^[^ ^35^, ^57^, ^58^ ^]^ and density‐driven flows^[^ ^40^, ^59^, ^60^ ^]^ do not significantly influence bubble growth in the parameter space considered in this study. This further shows that the role, if any, played by weaker, density and concentration‐driven flows may be overpowered by flows generated due to bubble coalescence and departure on electrodes with next‐neighbor bubble coalescence.

### 2.5 Electrode Coverage

**Figure 5:**
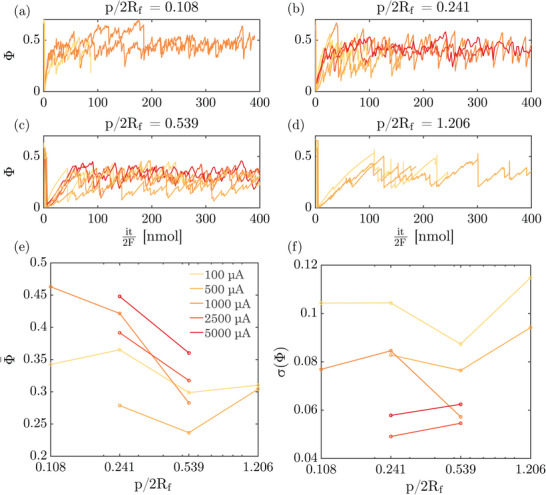
a–d) The temporal variation of Φ, the coverage of the observed area of the electrode by bubbles for different currents and pit‐spacings is plotted against the number of moles of hydrogen evolved at the electrode. The multiplication of the experimental time *t* by the molar rate of hydrogen production at the electrode *i*/2*F* enables the comparison of experiments conducted at different currents on the same scale. e,f) The mean (e) and standard deviation (f) of Φ (from plots (a–d)) are shown as functions of current and pit spacing. The x‐axis in (e) and (f) is log‐spaced for better readability but does not hold physical significance to the interpretation. Note that the different colors of lines in all six plots in this figure represent different applied currents as shown in the common legend displayed in (e).

Electrode coverage Φ represents the portion of the electrode that is shadowed by bubbles. The temporal variation of Φ during experiments at different currents and pit‐spacing rations is shown in **Figure** **5**a–d. Here, Φ is plotted against the moles of hydrogen evolved at the electrode which increases linearly with time for a constant current as *it*/2*F*. Plotting against the number of moles instead of time allows experiments driven by different currents to be compared on the same scale.

Figure 5a–d shows that the frequency and magnitude of the fluctuations in Φ is different for different pit‐spacings. This is further explored in Figure 5e,f where the mean and the standard deviation of Φ are plotted for different currents and pit‐spacings. The mean electrode coverage decreases with increasing pit‐spacing as long as *p*/2*R*_*f*_ < 1. This is because Φ represents a two‐dimensional representation of the bubble population on the electrode at any given instant in time. As *p*/2*R*_*f*_ increases, the departure radius also increases (see Figure S6, Supporting Information) leading to the formation of fewer but larger bubbles which is reflected in a lower two‐dimensional coverage. In our experiments, Φ does not represent the loss in electrode area due to bubbles since the bubbles are not hemispherical caps but remain spherical and pinned to the hydrophobic cavities throughout their lifetime. Rather, Φ in our system can be taken to be a measure of the constriction of ion conduction pathways at the electrode‐electrolyte interface. Thus, an increase in Φ is expected to increase the Ohmic resistance between the working and the counter electrode.

It can be seen that Φ is the largest for *p*/2*R*_*f*_ = 0.108. Thus, electrodes with the smallest pit spacing experience the greatest constriction of ion conduction pathways. This contrasts the supersaturation lowering effect discussed in the previous section. The beneficial supersaturation lowering effect was strongest for substrates with smaller pit spacings. This interplay between the negative effect of increased Ohmic losses due to coverage and the concentration lowering effect which alleviates the concentration overpotential requires further study and optimization.

## 3 Conclusion

A novel electrode architecture consisting of a silicon substrate with ordered arrays of hydrophobic cavities was designed and fabricated. The hydrophobic cavities are crowned by an oxide rings which rise above the silicon surface and aid the pinning of bubbles to the hydrophobic cavities. The pit‐separation distance was varied and its effect on bubble growth and departure were studied.

Broadly, two cases were expected depending on the pit‐to‐pit distance (p). On substrates where pits were closer than the Fritz departure radius, bubbles were expected to coalesce and depart at half the distance between the pits. On substrates where pits were spaced farther apart than the Fritz departure radius, bubbles were expected to grow without coalescence with neighboring bubbles and depart at the Fritz radius.

Experiments confirm that ∼83.4% of all bubbles in the study departed within the expected departure radius (*R*_*e*_) on all substrates. Notably, the nucleation of bubbles at non‐pit locations due to electrode roughness, the temporal asynchronicity in nucleation of bubbles and the deactivation of hydrophobic cavities lead to asymmetric bubble coalescence and wide distributions of bubble departure radii. Further, it was shown that for substrates with pit spacing ratios *p*/2*R*_*f*_ = 0.108, bubbles grew up to four times larger than the *R*_*e*_. This was attributed to the small size of bubbles on these substrates. The coalescence of these small bubbles was not sufficient to overcome the force of surface tension pinning them to the hydrophobic cavities.

It was also shown that the volumetric bubble growth rate $\dot{V}$ increased with increasing current (see Figure S9, Supporting Information) and increasing pit spacing ratios. The effect of current was no longer visible upon normalizing $\dot{V}$ with the volumetric rate of hydrogen generation at the electrode. Furthermore, the lower volumetric growth rates on substrates where pits were closely spaced indicates a decrease in the effective supersaturation of the electrolyte with decreasing pit spacing. Since bubbles on these electrodes departed at smaller sizes, for the same applied current, the concentration boundary layer was disrupted more frequently. This supports the view that the dominant mass transfer phenomena on gas‐evolving electrodes are coalescence‐induced flows. Further research needs to be conducted to determine the influence of concentration‐ and temperature‐driven flows on electrodes with next‐neighbor bubble coalescence.

Finally, a treatment of the 2D bubble coverage Φ, showed that the coverage increased with decreasing pit spacing ratios. This is expected to lead to an increase in the Ohmic resistance between the working and counter electrodes. This negative effect of decreasing pit spacing acts counter to the positive supersaturation alleviating effect. Future studies may focus on the optimization of these competing effects as a means to increase the efficiency of gas‐evolving electrochemical reactions.

The novel electrode architecture in this study, with arrays of preferential bubble nucleation sites, represents a significant advance over studies focusing on bubble evolution at microelectrodes. This electrode architecture with arrays of hydrophobic cavities has the potential to bridge the gap between microelectrode studies where single bubble evolution is studied in the absence of coalescence with neighboring bubbles, and macroscale studies where stochastic bubble evolution is studied. The results of this study warrant further investigation into the complex mass transfer phenomena that accompany electrolytic bubble growth in the presence of next‐neighbor coalescence.

Numerical modeling or direct experimental measurement of natural convection and dissolved gas concentrations during bubble growth are needed to elucidate the origin of the growth‐rate enhancement effect. Future studies may focus on the role played by parameters such as the electrolyte composition, electrolyte flow, electrode orientation and electrostatic interactions at the three‐phase boundary. Systems with preferential nucleation spots may be leveraged to utilize bubble coalescence as a passive method to control the size of bubbles in practical applications. This requires a deeper investigation into the physical mechanisms that underpin gas entrapment in hydrophobic cavities as well as deactivation mechanisms.

## 4 Experimental Section

### Electrode Fabrication

**Figure 6:**
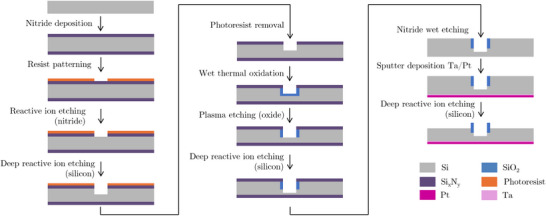
An illustration of the electrode microfabrication steps. First, a 2 μm non‐stoichiometric Si_x_N_y_ layer is deposited on to the highly‐doped silicon substrate. Then, a positive photoresist is spun onto the top‐face of the wafers and developed with a mask which transfers the areas of the hydrophobic cavities. Following this, the nitride layer is etched with reactive ion etching and the patterned nitride layer is used as a hard mask for subsequent steps. Next, a 2 μm pit is etched into the silicon using the deep reactive ion etching (DRIE). This is followed by the growth of a thermal 1 μm thick thermal oxide layer in the walls of the exposed silicon. Next, the oxide at the bottom of the pit is directionally etched using DRIE. The exposed silicon at the bottom of the pit is then etched until a depth of 10 μm and a black silicon surface is formed at the bottom to improve hydrophobicity. The nitride layers are then removed through wet etching in phosphoric acid. Subsequently, a 100 nm platinum back contact is sputter deposited with a 10 nm tantalum adhesion layer. Finally, the entire exposed silicon surface is etched 1 μm using DRIE. This results in the final pit geometry with a SiO_2_ ring protruding from the electrode surface.

**Figure** **6** shows an illustration of the fabrication steps that were employed to produce the electrodes used in this study. Detailed process parameters for sample fabrication are provided in Section S6 (Supporting Information). The samples were fabricated from boron‐doped silicon wafers (Okmetic; diameter: 100 mm thickness: 525 μm, resistivity: 0.01 – 0.025 Ωcm). First, a 2 μm thick non‐stoichiometric silicon rich nitride Si_x_N_y_ layer is deposited on the silicon wafer by means of LPCVD (Tempress Systems). Then a positive photoresist (Olin OiR 908‐35) is spun onto the wafer and exposed with a mask that transfers the areas corresponding to the hydrophobic cavities into the photoresist after developing. This is followed by the reactive ion etching of the Si_x_N_y_ layer (PlasmaTherm 790) and the deep reactive ion etching of the underlying silicon bulk for 2 μm (SPTS Pegasus). After stripping the photoresist, wet thermal oxidation (Tempress Systems) is used to locally oxidize the exposed silicon surfaces and grow a 1 μm thick layer of silicon oxide.

A directional plasma etching step (PlasmaTherm 790) is then used to remove the silicon oxide at the bottom of the 2 μm deep pit, leaving the oxide intact on the side walls of the shallow pits (see Figure S7, Supporting Information). Subsequently the exposed silicon is etched by means of deep reactive ion etching (SPTS Pegasus) until a depth of 10 μm and a second plasma etching step in the same machine is used to create black silicon at the bottom of the pit (see Figure S7, Supporting Information). During both etching steps the Si_x_N_y_ layer acts as a hard mask, thus requiring the initial large thickness of the layer.

The Si_x_N_y_ layer is then selectively etched in hot phosphoric acid to strip the Si_x_N_y_ layer from both sides of the wafer. On the backside of the wafer a 100 nm thick layer of platinum is deposited on top of a 10 nm adhesion layer of tantalum using an in‐house built RF sputtering tool (T'COathy). This provides a back‐contact terminal for the electrode. Finally, the entire silicon surface on the front side is etched for 1 μm by deep reactive ion etching (SPTS Pegasus). This leaves the silicon oxide ring at the side wall of the pits intact and still attached to the bulk of the wafer in the form of cups at the rim of the pits that rise above the etched silicon surface. As a last step, the wafer is diced into 10 × 10 mm^2^ squares.

## Conflict of Interest

The authors declare no conflict of interest.

## Supporting information

Supporting Information

Supplemental Video 1

## Data Availability

The data that support the findings of this study are available in the supplementary material of this article.
