# Supplementary material for: Electrolytic Bubble Coalescence on Hydrophobic Cavity Arrays Determines Departure Radius and Lowers Electrolyte Supersaturation
Source: Small. 2025 Sep 18;21(44):e05728. doi: 10.1002/smll.202505728 (PMC12590528; doi:10.1002/smll.202505728)
Supplement: Supplementary file 1 — Supporting Information [file SMLL-21-e05728-s001.pdf]

NANO · MICRO

small

Supporting Information

for *Small*, DOI 10.1002/smll.202505728

Electrolytic Bubble Coalescence on Hydrophobic Cavity Arrays Determines Departure Radius and Lowers Electrolyte Supersaturation

Akash Raman, Stefan Schlautmann, Han Gardeniers and David Fernández Rivas**

Supplementary Information to "Electrolytic bubble coalescence on hydrophobic cavity arrays determines departure radius and lowers electrolyte supersaturation"

Akash Raman*, Stefan Schlautmann, Han Gardeniers, David Fernández Rivas*
Mesoscale Chemical Systems Group, MESA+ Institute for Nanotechnology, Faculty of Science and Technology, University of Twente, P.O. Box 217, 7500 AE Enschede, The Netherlands.
* *E-mail: a.raman@utwente.nl, d.fernandezrivas@utwente.nl*

S1 Current density

i (µA) → $p/2R_f$ ↓	100	500	1000	2500	5000
0.108	1.1260	5.6298	11.2597	28.1492	56.2984
0.241	1.1138	5.5691	11.1382	27.8456	55.6911
0.539	1.1116	5.5581	11.1162	27.7904	55.5808
1.206	1.1112	5.5561	11.1123	27.7807	55.5614

Table SI 1: Current densities in mA/cm^2 for combinations of pit spacing and applied current considered in this study.

The hydrophobic cavities on the substrates occupy a finite area that is not available for hydrogen evolution. The number of pits on the silicon electrode depends on the pit spacing. Table SI 1 lists the current densities of corresponding to different applied currents for each pit spacing considered in this study.

S2 Image processing and analysis

All image processing and data analysis was performed on MATLAB R2022b and depended on functions from the Image Processing Toolbox (v11.6) and the Parallel Computing Toolbox (v7.7). The data processing steps are listed here.

1. *Measuring optical resolution:* The optical resolution of each image sequence was calculated in µm/px by measuring the known pit-to-pit distance using FIJI[1].

2. *Background subtraction:* The first image of each experimental sequence was subtracted from each subsequent image and the background-subtracted images were binarized using Otsu's method[2] and the proportion of white pixels in the binarized image was measured and stored as the coverage, ϕ.

3. *Bubble detection:* Bubble were detected using a two-stage circular Hough transform[3,4]. The upper and lower bounds for the Hough circle detection step were specified in pixels as $1.2\times R_f$ and R_p respectively, where $R_p = 5\mu m$ is the pit radius and R_f is the Fritz radius. These wide radius bounds for the Hough algorithm were necessitated by the range of bubbles sizes expected in every frame. The entire image analysis pipeline was sped up using parallel processing.

4. *Hough artefacts removal:* Bubbles that were contained within larger bubbles were detected by comparing the radii and centres of all detected bubbles in every frame. Such bubbles were artefacts arising from the wide radius bounds specified for the Hough transform.

5. *Bubble stitching:* The radii and centres of bubbles was collated across successive frames and effectively grouped by the position of their nucleation site. First, all bubbles in the first frame were marked as 'active' using a flag variable. Then, the positions of bubbles marked active was compared against the centres of bubbles in the subsequent frame. A threshold of 10 pixels was adopted to overcome variability from the circle detection stage. If no corresponding bubbles were found at the same location of an active bubble, it was marked 'inactive' and stored in a separate array. New bubbles detected in the subsequent frame were marked 'active' themselves. This process was continued for each frame and bubbles appearing in multiple frames were stitched together i.e., their data arrays were concatenated.

6. *Fixing breaks in bubble curves:* Due to the top-down imaging used in the experimental setup, the departure of nearby bubbles could cause the Hough transform to miss bubbles in the detection stage. These inconsistencies would result in spurious breaks in the bubble growth curves. These were stitched by comparing the locations and radii of bubbles within a four frame window.

7. *Separating successive bubbles:* At the end of the last two steps, long arrays of bubbles grouped by location were obtained. Then, individual bubble departures within these bubble successions were detected by identifying drops in bubble radii ($\Delta R_b \leq -5$ μm) and each bubble was stored separately.

8. *Further filtering:* The script occasionally detected pits as circles leading to sections of bubble growth curves where bubbles appeared to not grow. These were removed as well as any bubbles that only appeared in four frames since these were typically erroneous bubbles that were not filtered by the previous steps.

9. *Estimating nucleation time:* The nucleation time, t_0 of each bubble was estimated through linear interpolation of the first 1.5 s of R_b^2 and t data points, where t is experimental time. The choice of the exponent of R_b was motivated by the slopes observed in the bubble growth curves (see Fig. 2).

   Note: This method of estimating the nucleation time does not work for bubbles that were formed as a result of coalescence. Bubbles formed due to coalescence are visible in Fig. 2 as growth curves that begin at $R_b \geq 20$ μm.

10. *Calculating bubble growth rate:* The growth rate of each bubble, β^3 was calculated as the slope of a linear fit for R_b^3 and t_b, where $t_b = t - t_0$ is the bubble lifetime.

S3 Preliminary substrate designs

Alternative fabrication routes and electrode designs were tested before the development of the oxide-cup designs but did not function as intended. These designs are briefly described here to guide future research and development.

The first version comprised of a sputter-deposited platinum electrode on the same highly-doped silicon wafer with hydrophobic cavities (without the oxide cups) etched into it. Preliminary experiments with these wafers lead to the generation of bubbles much larger than their Fritz radius, $R_f \sim 382$ μm for pit radius $r_p = 5$ μm (see Eq. 1). Optical microscopy images revealed that the hydrophobic cavities were entirely visible through the bubbles which in combination with the larger departure radii confirmed that the bubbles were not pinned at the pit and were instead adhering to the platinum surface.

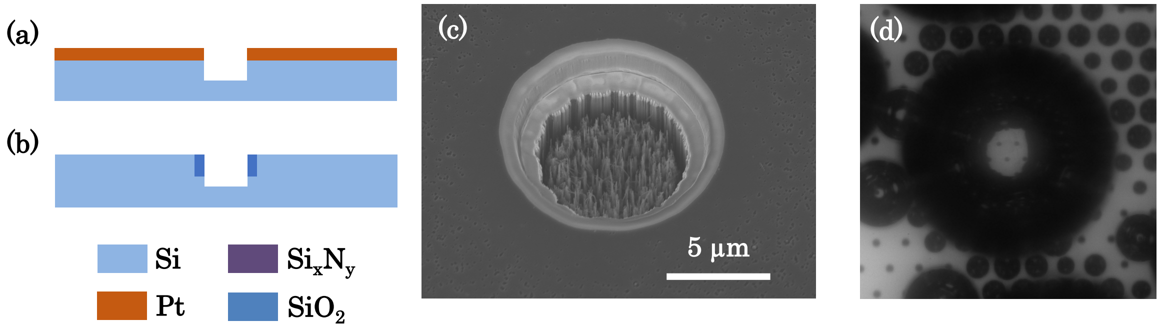

Figure SI 1: (a) and (b) depict alternative electrode designs that were tested before the adoption of the oxide-cup electrodes described in the main text. In (a) the pits are etched through a platinum layer whereas (b) shows a design where an oxide ring surrounds the pit walls. Image (c) shows a scanning electron microscope image of a single pit with an oxide ring around the pit. This corresponds to design (b). (d) A closeup view of a bubble in an experiment with substrates with the oxide rings around the pits (design (b)). Multiple pits are visible through the apex of the bubble. This would normally not be possible if the bubble was pinned to a pit.

It was then posited that a thin ($\sim$ 0.5–1.5 μm wide) silicon-oxide ring around the pits would solve this issue by providing a material discontinuity between the platinum electrode and the pit. This however raised the challenge of aligning the mask for the oxide rings with the pits with a precision of the order of less than a micron. Optical alignment of mask layers with this precision was found to be challenging which lead to the development of a self-aligned oxide ring process. However, since this process involved the use of a metal-free furnace, the platinum electrode layer had to be discarded, leaving a silicon electrode. Unfortunately, the problem of bubbles growing larger than R_f persisted.

Finally, substrates with the oxide cups were serendipitously created when a batch of wafers was over-etched during the deep reactive ion etching to create the pits. This occurred because the Si_xN_y hard masking layer was too thin and was etched away leaving the oxide cup. It was found that in the presence of the oxide cups, bubbles remained pinned to the hydrophobic cavities and departed at the expected radius in the absence of coalescence. Thus, this fabrication process was standardized and adopted for the study.

Fig. S3 shows two previous electrode designs that were fabricated. In both cases, bubbles did not pin to the pits as expected. Bubbles were observed depinning from the pit - with their contact line advancing along the electrode surface. This was confirmed when multiple bubbles were clearly visible through the apex of the bubbles and they grew to radii much larger than the Fritz radius associated with the pits.

The adoption of the oxide-cup design resolved the issue of bubble depinning. As seen in the main text, the bubble departure radii were in line with expectations.

S4 Representative bubble growth curves

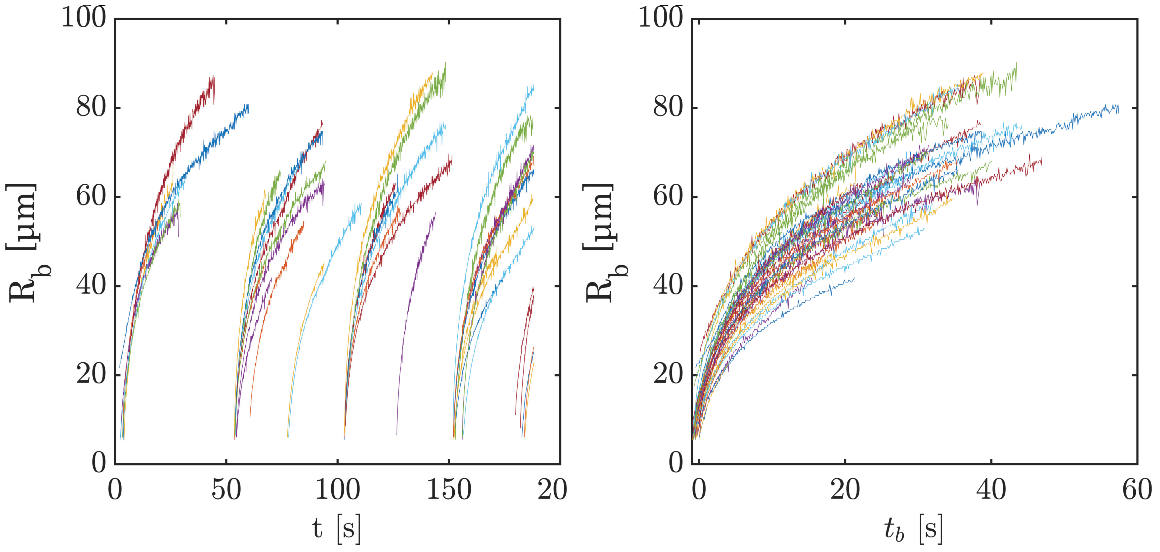

Figure SI 2: The bubble growth curves for a single representative experiment performed with a substrate with pit spacing corresponding to $p/2R_f = 0.241$ driven by an electrolysis current of 100 μA. Bubble radii R_b are plotted against the experimental time t in the plot on the left and against bubble lifetime t_b on the plot on the right. No additional smoothing functions are used to process the bubble growth curves. The colors of the curves do not have a physical relevance in this plot.

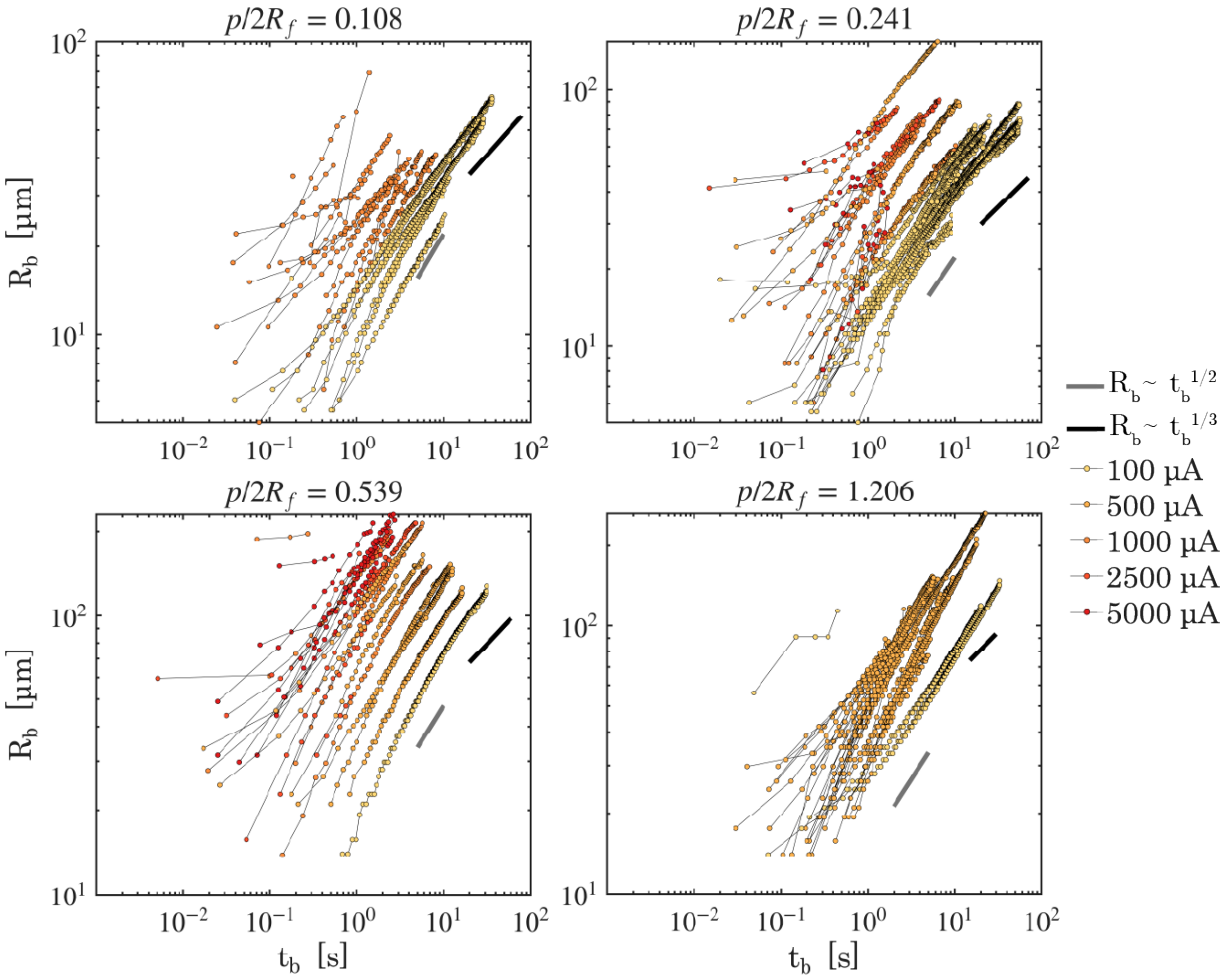

Figure SI 3: Representative bubble growth curves of bubbles i.e., bubble radii R_b plotted as functions of bubble lifetime t_b, are shown on a log-log scale. Growth curves beginning at $R_b \geq 20$ µm represent bubbles resulting from the coalescence of preceding bubbles. The gray line indicates the slope corresponding to $R_b \propto t_b^{1/2}$ and the black line indicates the slope corresponding to $R_b \propto t_b^{1/3}$.

S5 Pit inactivation and non-pit bubbles

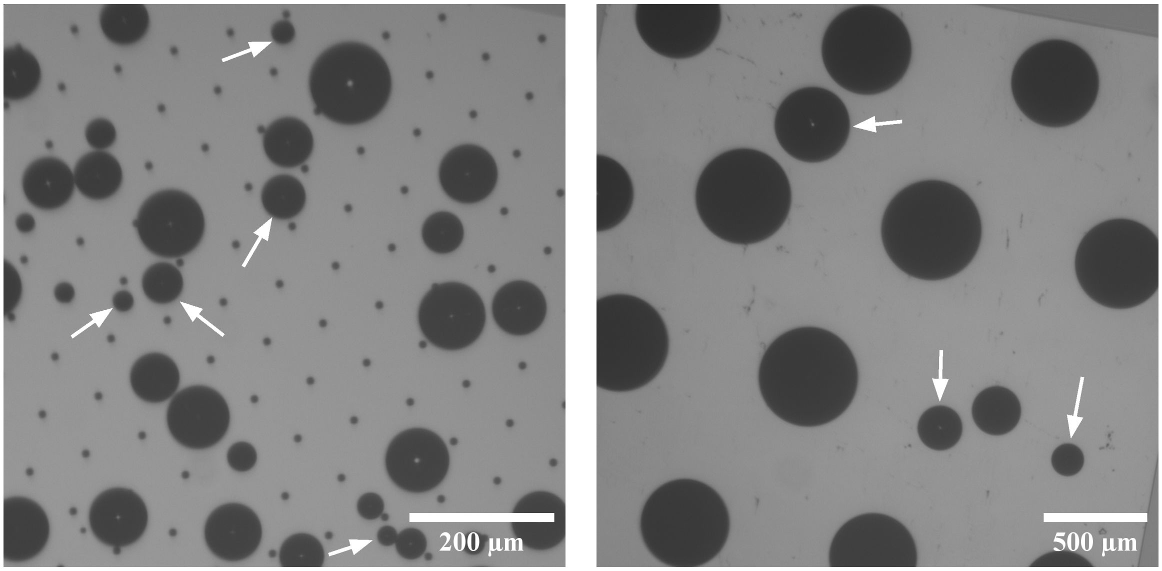

Figure SI 4: These experimental images of substrates corresponding to $p/2R_f =$ 0.108 and 1.206 show deviations from the expected behavior. Bubbles that nucleate at locations other than the hydrophobic cavities are indicated with white arrows. In the image on the left, several inactive pits which no longer act as bubble nucleation points can be seen.

S6 Fabrication process parameters

All processes have been performed on intact boron-doped silicon wafers (Okmetic; diameter: 100 mm thickness: 525 μm, resistivity: 0.01 – 0.025 Ω cm).

1. LPCVD for 2 μm non-stoichiometric silicon rich nitride deposition:
   Tempress Systems, 850 °C, 150 mTorr, SiH_2Cl_2: 72 sccm, NH_3: 22 sccm, N_2: 150 sccm, 270 min

2. Photoresist spin-coating: Olin OiR 908-35, 4000 rpm, 30 s
   Spin ramp: 500 rpm/s
   Spin speed: 4000rpm
   Spin time: 30s
   Soft bake: 120s at 95 °C

3. Photoresist exposure: EVG 620 mask aligner
   Mercury arc lamp, near UV range: 350 - 450 nm, intensity: 12 mW/cm2
   Contact mode: hard contact
   Exposure time: 9s

4. Photoresist development: Olin OPD 4262, 60 s dip development

5. Reactive ion etching of Si_xN_y:
PlasmaTherm 790 parallel plate reactive ion etching system
Etching conditon: CHF_3: 100 sccm, O_2: 12 sccm, 40 mTorr, 250W
Etching time: 60 min

6. Deep reactive ion etching (Bosch process) of 2 μm of silicon:
SPTS LPX Pegasus, plate temperature 20 °C, 20 Torr He pressure
Deposition: 0.6 s, C_4F_8: 150 sccm, inductively coupled plasma 2000 W
Etching: 1.75 s, SF_6: 275 sccm, inductively coupled plasma 2200 W, capacitively coupled plasma 20 W
Total cycles: 6

7. Photoresist stripping: PVA TePla 360 Pre-heat: Ar flow: 600 sccm, pressure: 0.6 mbar, power: 1000 W, time: 10 min Ashing: O_2 flow: 360 sccm, Ar flow: 160 sccm, pressure: 0.6 mbar, power: 800 W, time: 1 hour

8. Local oxidation to produce 1 μm SiO_2:
Tempress Systems, atmospheric wet oxidation 1150 °C, 135 min

9. Directional plasma etching of oxide at bottom of pits:
PlasmaTherm 790, CHF_3: 100 sccm, O_2: 5 sccm, 40 mTorr, 250 W, etching time: 30 min

10. Deep reactive ion etching (Bosch process) of 10 μm of silicon at pit bottom:
SPTS LPX Pegasus, plate temperature 20 °C, 20 Torr He pressure
Deposition: 0.6 s, C_4F_8: 150 sccm, inductively coupled plasma 2000 W
Etching: 1.75 s, SF_6: 275 sccm, inductively coupled plasma 2200 W, capacitively coupled plasma 20 W
Total cycles: 30

11. Deep reactive ion etching (Bosch process) to create black silicon at pit bottom:
SPTS LPX Pegasus, plate temperature 20 °C, 20 Torr He pressure
Deposition: 0.6 s, C_4F_8: 150 sccm, inductively coupled plasma 2000 W
Etching: 1.75 s, SF_6: 40 sccm, inductively coupled plasma 2200 W, capacitively coupled plasma 20 W
Total cycles: 128

12. Nitride etching: Wet chemical etching, 5 hours in 85 % H_3PO_4 at 180 °C

13. Back contact sputtering:
T'COathy (in-house RF sputtering tool), base pressure: 7e-7mbar, process pressure: 6.6e-3 mbar, 200 W
10 nm Ta adhesion layer: 1 min
100 nm Pt: 4 min

14. Deep reactive ion etching (Bosch process) of 1 μm silicon:
SPTS LPX Pegasus, plate temperature 20 °C, 20 Torr He pressure
Deposition: 0.6 s, C_4F_8: 150 sccm, inductively coupled plasma 2000 W

Etching: 1.75 s, SF_6: 275 sccm, inductively coupled plasma 2200 W, capacitively coupled plasma 20 W Total cycles: 3

15. Wafer dicing: DISCO DAD3220, NBC-Z-2050 blade, dicing speed 10 mm/s, 10×10 mm^2 grid

S7 Bubble departure radius data

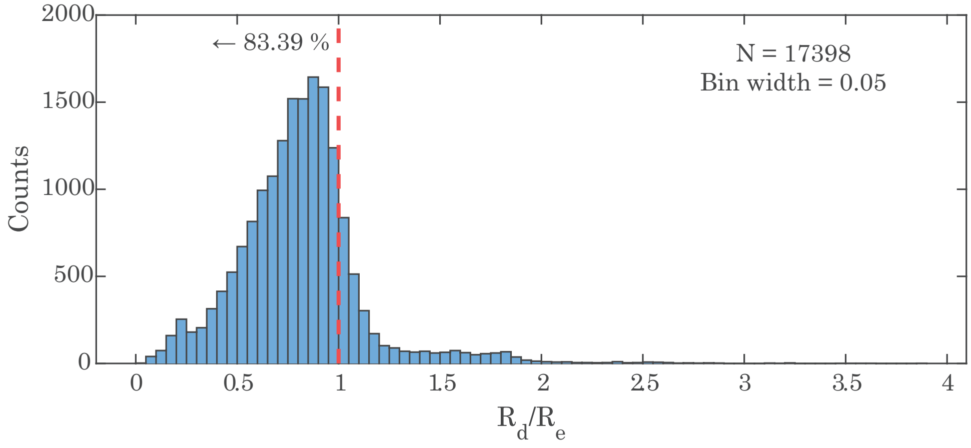

Figure SI 5: Histogram of the normalized bubble departure radii of all bubbles observed across all experiments. Here, R_d are normalized by the expected bubble radius R_e. The theoretical or ideal case is where each bubble departs at $R_d = R_e$. It is found that 83.39 % of all bubbles observed depart at a radius less than R_e.

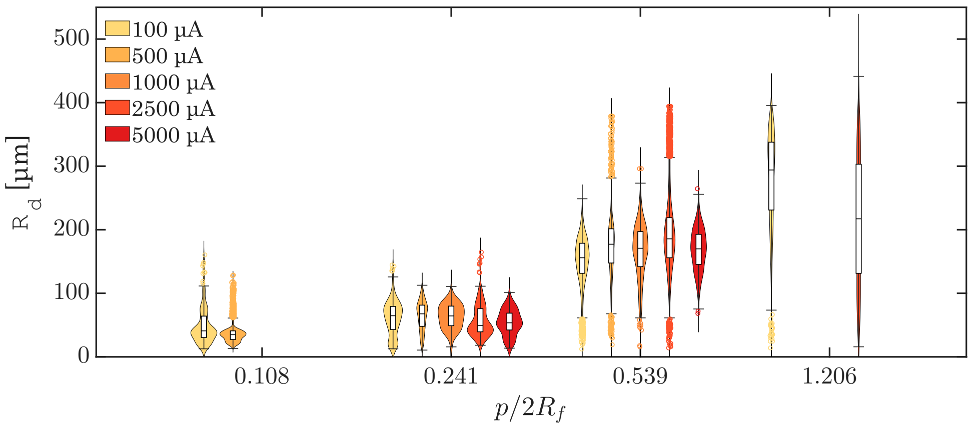

Figure SI 6: Violin and box plots of the departure radii, R_d of bubbles growing on substrates. Data for bubbles driven by different currents are shown by the color of the kernel density estimation curves and grouped by the pit-separation ratios $p/2R_f$ of the substrates, where p is the distance between adjacent pits and R_f is the Fritz departure radius. The bandwidths were estimated using the normal-approximation method. The white boxes represent the middle 50 % of each dataset and show the sample median as a horizontal line. The whiskers represent the extent of data and outliers are plotted as circles.

S8 SEM image of hydrophobic cavity

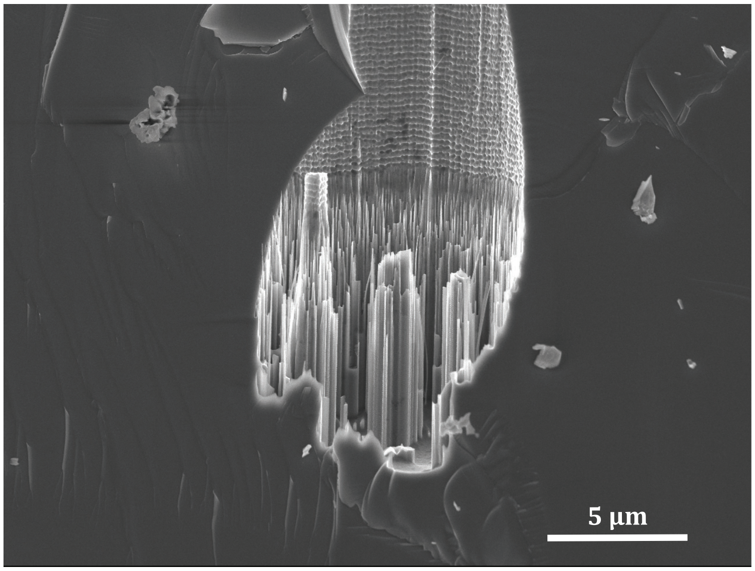

Figure SI 7: An SEM image showing a zoomed-in view of the internal structure of a hydrophobic cavity on an electrode used in the study. The electrode was intentionally snapped to make it possible to image the internal walls of the pit. Scallops produced during the deep reactive ion etching of the pits can be seen on the side walls. The needle-like structures at the bottom of floor of the pit are the black silicon. Together, these two features impact hydrophobicity to the pits and enable them to trap gas and facilitate bubble nucleation.

S9 Number of pits in field of view

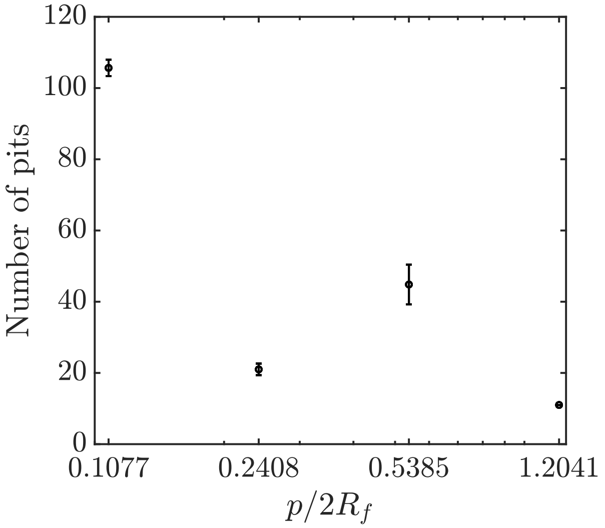

Figure SI 8: The number of pits in the field of view during experiments with substrates with different pit spacing ratios $p/2R_f$; where p is the distance between two adjacent pits on the substrates and R_f is the theoretical departure radius or the Fritz radius. Images for substrates with $p/2R_f = 0.108$ and 0.241 were recorded at 5× magnification and images for substrates with $p/2R_f = 0.539$ and 1.206 were recorded at 2× magnification. The error bars represent two standard deviations about the sample mean.

S10 Fritz radius calculations

$$\sigma = \sigma_0 + c\frac{d}{dc}(\Delta\sigma)$$

$$R_f = \left(\frac{3\sigma r_p}{2\Delta\rho g}\right)^{\frac{1}{3}}$$

Radius of pit	r_p	10 μm
Surface tension in 0.1 M H_2SO_4	σ	72.414 mNm^{-1} [5]
Surface tension coefficient for H_2SO_4	$d(\Delta\sigma)/dc$	0.44[5]
Surface tension of water	σ_0	72.37 mNm^{-1}[5]
Concentration of H_2SO_4	c	0.1 M
Density difference	$\Delta\rho$	997 kg/m^3
Fritz radius	R_f	381.50 μm

Table SI 2: Values of physical parameters used in the paper along with the calculated Fritz radius.

S11 Sample sizes

i (μA)	p	Sample size
100	0.1077	216
100	0.2408	382
100	0.5385	2544
100	1.2041	1971
500	0.1077	0
500	0.2408	259
500	0.5385	3313
500	1.2041	1185
1000	0.1077	3272
1000	0.2408	719
1000	0.5385	1509
1000	1.2041	0
2500	0.1077	0
2500	0.2408	158
2500	0.5385	792
2500	1.2041	0
5000	0.1077	0
5000	0.2408	137
5000	0.5385	941
5000	1.2041	0

Table SI 3: A tabular view of the number of bubbles recorded for each combination of current and pit spacing ratio in this study.

S12 Volumetric bubble growth rates

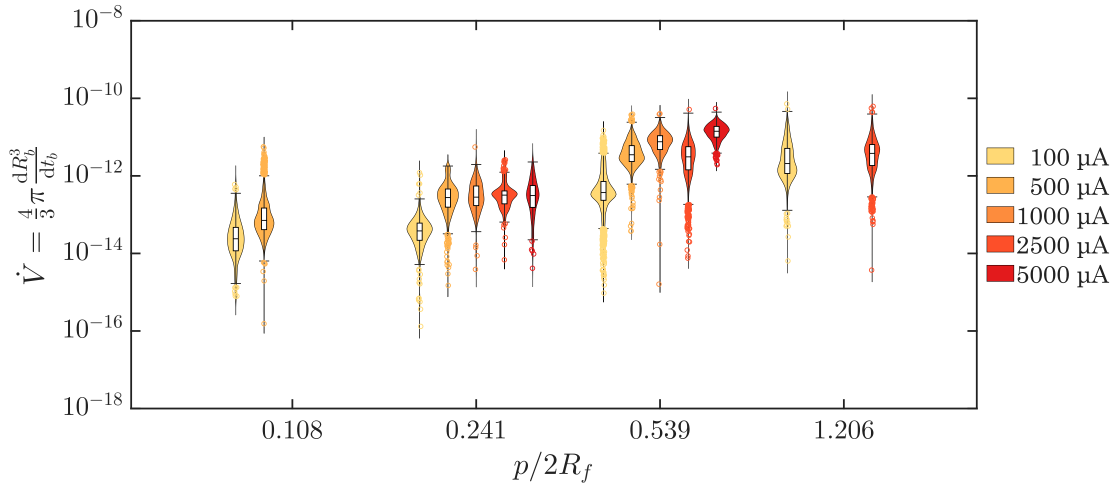

Figure SI 9: Violin and box plots of $\dot{V}$, the bubble growth coefficient. Data for bubbles driven by different currents are shown by the color of the kernel density estimation curves and grouped by the pit-separation ratios $p/2R_f$ of the substrates, where p is the distance between adjacent pits and R_f is the Fritz departure radius. The bandwidths were estimated using the normal-approximation method. The white boxes represent the middle 50 % of each dataset and contain the sample median shown as a horizontal line. The whiskers represent the extent of data falling within 1/5 × the interquartile range and outliers are plotted as circles. It can be seen that β' increases with increasing pit-separation ratios.

References

[1] J. Schindelin, I. Arganda-Carreras, E. Frise, V. Kaynig, M. Longair, T. Pietzsch, S. Preibisch, C. Rueden, S. Saalfeld, B. Schmid, J.-Y. Tinevez, D. J. White, V. Hartenstein, K. Eliceiri, P. Tomancak, A. Cardona, *Nat Methods* **2012**, *9*, 7 676.

[2] N. Otsu, *IEEE Trans. Syst. Man Cybern.* **1979**, *9*, 1 62.

[3] T. J. Atherton, D. J. Kerbyson, *Image and Vision Computing* **1999**, *17*, 11 795.

[4] H. K. Yuen, J. Princen, J. Dlingworth, J. Kittler, In *Procedings Alvey Vis. Conf. 1989*. Alvey Vision Club, Reading, **1989** 29.1–29.6.

[5] P. K. Weissenborn, R. J. Pugh *184*, 2 550.
